# Sex differences in the association of uric acid to HDL-cholesterol ratio (UHR) with subclinical cardiovascular damage

**DOI:** 10.1186/s13293-026-00956-z

**Published:** 2026-07-22

**Authors:** Mariangela Rubino, Angela Palummo, Mattia Massimino, Carolina Averta, Stefania Belviso, Asia Servello, Francesco Maria Brisinda, Alberto Cosenza, Angela Sciacqua, Maria Perticone, Francesco Perticone, Elettra Mancuso, Gaia Chiara Mannino, Francesco Andreozzi

**Affiliations:** 1 Department of Medical and Surgical Science, University Magna Graecia of Catanzaro, Viale Europa, Catanzaro, 88100 Italy; 2 Department of Experimental and Clinical Medicine, University Magna Graecia of Catanzaro, Viale Europa, Catanzaro, 88100 Italy; 3 AO Renato Dulbecco University Hospital of Catanzaro, Catanzaro, 88100 Italy; 4 Department of Science of Health, University Magna Graecia of Catanzaro, Viale Europa, Catanzaro, 88100 Italy

**Keywords:** Uric Acid to HDL-Cholesterol Ratio (UHR), Subclinical Cardiovascular Damage, Left Ventricular Mass Index (LVMI), Carotid Intima-Media Thickness (c-IMT), Sex-Specific Biomarker

## Abstract

**Background:**

Cardiovascular diseases remain the leading cause of global mortality, with risk increasing significantly in women post-menopause. Identifying accessible biomarkers for subclinical risk stratification is a clinical priority. The uric acid to HDL-cholesterol ratio (UHR) has emerged as a composite marker reflecting metabolic burden and reduced vascular protection. This study investigates the independent association between UHR and subclinical organ damage, exploring sex-specific differences.

**Methods:**

We analyzed 1,198 Caucasian subjects. Subclinical cardiovascular damage was evaluated via carotid intima-media thickness (c-IMT) and left ventricular mass index (LVMI). Multivariate linear regression and formal interaction analyses were employed to assess the independent association of UHR with organ damage, adjusting for traditional cardiometabolic risk factors.

**Results:**

UHR correlated significantly with adiposity, insulin resistance, blood pressure, and lipid profiles. In the total population, UHR was independently associated with both c-IMT (β = 0.093, *p* = 0.003) and LVMI (β = 0.194, *p* < 0.0001). A significant sex-UHR interaction was observed for LVMI (*p* = 0.024). Sex-stratified analyses revealed that UHR was linked to LVMI in women, particularly those postmenopausal, but not in males. In postmenopausal women, UHR remained an independent determinant of LVMI (β = 0.164, *p* = 0.033).

**Conclusions:**

UHR is independently associated with subclinical cardiovascular damage, displaying a sex-specific relationship with myocardial remodeling after menopause. As an inexpensive and easily accessible biomarker, UHR may represent a practical tool to combat clinical inertia and improve the early selection of postmenopausal women warranting targeted diagnostic workups.

**Supplementary Information:**

The online version contains supplementary material available at https://doi.org/10.1186/s13293-026-00956-z.

## Abstract (plain language summary)

Heart disease is the leading cause of death worldwide, and women face a particularly sharp rise in risk after menopause. To prevent cardiovascular events, it is vital to find simple ways to spot early, hidden signs of trouble before a person even feels sick. In our study, we looked at an accessible tool called the Uric Acid to HDL-Cholesterol Ratio (UHR). This is a simple value that doctors can easily calculate at no extra cost using two common markers found in routine blood tests: uric acid and HDL (“good”) cholesterol.

We studied nearly 1,200 individuals to see if a high UHR score was associated with early, subclinical damage to the heart muscle and the arteries in the neck. Our results showed that while a higher score is generally linked to poorer artery health across the entire population, it has a very specific meaning for women. Indeed, the link between this blood marker and an enlarged heart is remarkably strong in women who have gone through menopause, whereas no such significant association was detected in men.

This discovery is important because it suggests that menopause triggers metabolic changes that make the female heart more vulnerable to certain types of stress. By using this simple blood test ratio as an early biochemical indicator, doctors may more easily recognize postmenopausal women who exhibit silent changes in their heart structure, allowing clinicians to facilitate early intervention and prioritize them for deeper diagnostic checkups. Because this calculation is inexpensive and easily accessible, it offers a highly practical way to help women protect their heart health as they age.

**Supplementary Information:**

The online version contains supplementary material available at https://doi.org/10.1186/s13293-026-00956-z.

## Highlights

UHR serves as a novel, sex-specific biomarker for identifying subclinical cardiovascular organ damage.

Elevated UHR is independently associated with both carotid wall thickening and increased left ventricular mass in a large Caucasian cohort.

Formal interaction analysis confirms that the impact of UHR on cardiac structure is affected by biological sex.

The association between UHR and myocardial remodeling is particularly prominent in postmenopausal women.

**Supplementary Information:**

The online version contains supplementary material available at https://doi.org/10.1186/s13293-026-00956-z.

## Introduction

Cardiovascular diseases (CVD) represent the leading cause of morbidity and mortality worldwide, accounting for approximately 17.3 million deaths each year, with projections reaching 25 million by 2030 [1]. Identifying reliable biomarkers capable of detecting cardiovascular risk at early, subclinical stages remains a major goal of clinical research, as improved risk stratification may allow timely preventive interventions.

Left ventricular hypertrophy (LVH), defined as an inappropriate increase in left ventricular mass (LVM) beyond compensatory hemodynamic requirements, is a strong and independent predictor of cardiovascular (CV) and all-cause mortality [2, 3]. Although elevated blood pressure is a key determinant of LVH, growing evidence indicates that neurohormonal and metabolic factors also play a substantial role in left ventricular remodeling [4, 5]. Similarly, arterial disease progresses through vascular wall thickening and stiffening driven by lipid accumulation, inflammation, and fibrosis [6]. In this context, carotid intima-media thickness (c-IMT) represents a well-established, noninvasive marker of subclinical atherosclerosis and a predictor of future CV events [7].

Uric acid (UA), the final product of purine metabolism, has been consistently associated with metabolic and CV disorders, including hypertension, chronic kidney disease, insulin resistance, and metabolic syndrome [8–12]. Elevated UA levels predict CV and all-cause mortality even within the normal range [13, 14]. Mechanistically, UA promotes oxidative stress, endothelial dysfunction, vascular inflammation, and smooth-muscle cell proliferation [15, 16], processes that directly contribute to both LVH and increased c-IMT [17].

Notably, sex-related differences significantly modulate UA metabolism and its CV impact [12]. Women typically exhibit lower UA levels, due to the uricosuric effects of estrogens; nevertheless, meta-analytic data report a stronger association between UA and adverse CV outcomes in women compared with men [18]. Moreover, inverse J-shaped relationships between UA levels and stroke incidence or CV mortality have been observed predominantly in female populations [19, 20], suggesting that UA-related pathways such as xanthine oxidase activity and oxidative stress may exert sex-specific CV effects modulated by hormonal status.

In parallel, high-density lipoprotein cholesterol (HDL-Chol) plays a central role in reverse cholesterol transport and vascular protection [21]. Reduced HDL-Chol levels are hallmarks of insulin resistance, metabolic syndrome, and atherosclerotic plaque development, even in individuals with low low-density lipoprotein-cholesterol (LDL-Chol) concentrations [22]. However, the relationship between HDL-Chol and CV outcomes is non-linear, following a J-shaped pattern where both very low and very high levels are associated with increased mortality risk [23]. These observations highlight the limitations of considering HDL-Chol as an isolated marker of CV protection.

Experimental and clinical evidence suggests a synergistic interaction between UA and HDL-Chol in atherosclerosis, as elevated UA levels may impair HDL functionality, promoting the formation of smaller and less protective HDL particles [24, 25]. To capture these combined metabolic and lipid-related effects, the uric acid to HDL-cholesterol ratio (UHR) has been proposed as a composite biomarker reflecting the interplay between oxidative burden and reduced vascular protection [26].

While recent studies have linked UHR to a wide spectrum of cardiometabolic conditions, including hypertension, diabetes, and chronic kidney disease [27–30], data remain limited regarding its impact on structural cardiac and vascular remodeling. Lately, the specific relevance of this biomarker in the postmenopausal setting has emerged; a large-scale analysis which demonstrated a robust, dose-dependent association between the UHR index and various obesity metrics specifically in postmenopausal women [31].

However, previous investigations have focused primarily on metabolic profiles rather than subclinical structural alterations, leaving it unexplored whether the UHR signal translates into target organ damage, and whether this relationship differs according to sex or menopausal status. In particular, no investigation has yet directly compared the relative strength of UHR versus blood pressure in relation to LVM index (LVMI) and c-IMT in postmenopausal women.

To address these gaps, this study aimed to investigate the independent association between UHR and subclinical CV organ damage, assessed by c-IMT and LVMI, in a large, well-characterized cohort. Based on the biological plausibility of sex-specific metabolic regulation and the emerging metabolic role of UHR after menopause, we specifically tested the hypothesis that the impact of UHR on structural organ damage displays significant heterogeneity according to sex and menopausal status.

## Materials and methods

### Study subjects

The study group consisted of 1198 Caucasian subjects enrolled in the CATAnzaro MEtabolic RIsk factors Study (CATAMERIS) [32, 33], a screening campaign for the assessment of cardio-metabolic risk factors conducted at the Department of Medical and Surgical Sciences of the University “Magna Graecia” of Catanzaro, Italy. Inclusion criteria were the presence of one or more cardiometabolic risk factors, including diabetes, hypertension, dyslipidemia, and overweight/obesity.

Exclusion criteria included chronic gastrointestinal diseases, chronic pancreatitis, history of malignant disease, alcohol or drug abuse, positive serology for hepatitis C virus (HCV) or hepatitis B surface antigen (HBsAg), and kidney or hepatic failure. Menopausal status was defined as the absence of spontaneous menses for at least 12 consecutive months prior to enrollment, in accordance with standard clinical criteria [34]. Women with amenorrhea due to surgical causes (bilateral oophorectomy) were also classified as postmenopausal. These subjects were included in subgroup analyses for post-menopausal women. Follicle-stimulating hormone (FSH) levels were not routinely measured for classification. Menopausal status and concomitant medications were self-reported at enrollment. A comprehensive and detailed pharmacological history was available for the entire study population (*N* = 1198). At the time of recruitment, none of the participants reported current use of allopurinol or other urate-lowering therapies, and no female participant reported the use of hormonal replacement therapy (HRT) or estroprogestins. Concomitant medications—including antihypertensive agents (RAAS inhibitors, diuretics, beta-blockers, calcium channel blockers, alpha-blockers, and vasodilators), lipid-lowering drugs (statins and fibrates), antithrombotic therapies (antiplatelets and anticoagulants), glucose-lowering drugs (metformin, other oral hypoglycemics, and insulin), and systemic therapies (thyroid hormones and corticosteroids) were systematically recorded at baseline and are fully detailed in Supplementary Table S1. Subjects using diuretics or other medications known to influence UA or HDL-Chol were accounted for and considered in sensitivity analyses.

All participants underwent a thorough physical examination; anthropometric, biochemical, and clinical data were collected at the recruitment visit. Blood sampling for laboratory determinations was performed in the morning after an overnight fast.

The study was approved by the Institutional Ethics Committees of the University “Magna Graecia” of Catanzaro. Written informed consent was obtained from each subject in accordance with the principles of the Declaration of Helsinki.

### Laboratory determinations

Glycated hemoglobin (HbA1c) was measured by high-performance liquid chromatography using a National Glycohemoglobin Standardization Program (NGSP)-certified automated analyzer (Adams HA-8160 HbA1C analyzer, Menarini, Italy). Triglycerides, total cholesterol (Tot-Chol), HDL-Chol concentrations were determined by enzymatic methods (Roche, Basel, Switzerland). Fasting plasma glucose (FPG) was measured by the glucose oxidation method (Beckman Glucose Analyzer II; Beckman Instruments, Milan, Italy). Fasting plasma insulin (FPI) concentration was measured using a chemiluminescence-based assay (Immulite^®^, Siemens, Italy).

### Ultrasound measurement of c-IMT and echocardiographic assessments

High-resolution B-mode ultrasound was used to measure c-IMT of the common carotid artery using an ATL HDI 3000 ultrasound system (Advanced Technology Laboratories, Bothell, WA) equipped with a 7.5 MHz transducer, as previously described [35]. To evaluate average c-IMT, six mean IMT values were obtained and averaged: three from the left and three from the right common carotid artery, measured at the distal segment approximately 1 cm before the bifurcation.

Echocardiographic assessments were performed by a single experienced examiner blinded to the clinical and laboratory data of participants. Tracings were obtained with patients in a partial left lateral decubitus position using a VIVID-7 Pro ultrasound machine (GE Technologies, Milwaukee, WI) with an annular phased array 2.5-MHz transducer. Only frames with optimal visualization of cardiac structures were analyzed. LVM was calculated using the Devereux formula [36]: LVM (g) = 0.8 × 1.04 × [(IVSd + LVDD + PWd)³ – LVDD³] + 0.6, where IVSd is interventricular septum thickness, LVDD is left ventricular end-diastolic diameter, and PWd is posterior wall thickness in diastole (all in cm). Left ventricular mass index (LVMI) was calculated by indexing LVM to body surface area (BSA), expressed in g/m².

### Calculations

UHR was calculated as the ratio between serum UA (mg/dL) and HDL-Chol (mg/dL). The homeostasis model assessment of insulin resistance (HOMA-IR) was calculated as:

HOMA-IR = (FPI×FPG)/22.5 [37]. LDL-Chol was calculated using the Friedewald formula: LDL-Chol = Tot-Chol – HDL-Chol – (Triglycerides/5); BSA was calculated by the Du Bois equation as BSA (m^2^) = 0.007184×height (cm)^0.725^× body weight (kg)^0.425^ [38].

### Statistical analysis

Continuous variables are expressed as mean ± standard deviation (SD), while categorical variables are reported as frequencies and percentages. Normality of data distribution was assessed using the Kolmogorov–Smirnov test. Variables with skewed distribution (triglycerides, FPI, and HOMA-IR) were logarithmically transformed before analysis. Differences between groups were evaluated using Student’s *t*-test for continuous variables and the χ² test for categorical variables. Pearson correlation coefficients were calculated to assess associations between continuous variables. Multivariable linear regression analyses were performed to evaluate the independent association between UHR and indices of subclinical cardiovascular organ damage (c-IMT and LVMI). Models were adjusted for relevant cardiometabolic covariates, including age, body mass index (BMI), systolic blood pressure (SBP), HOMA-IR and LDL-Chol. Standardized regression coefficients (β) were reported to allow comparison of effect sizes. To assess the robustness of our findings against potential confounding by medication use, sensitivity analyses were performed by explicitly evaluating a statistical Model (Model 2) including covariates for the use of medications for hypertension and dyslipidemia, both therapies being able to affect UA and HDL-Chol levels. To formally test whether the association between UHR and c-IMT or LVMI differed by sex, a General Linear Model (GLM) including a sex × UHR interaction term was constructed. Based on the interaction results, subsequent analyses were stratified by sex and, among women, by menopausal status. A two-sided p-value ≤ 0.05 was considered statistically significant. Full model diagnostics, including stepwise selection steps, Variance Inflation Factors (VIF), and analyses for the male and pre-menopausal subgroups, are provided in Supplementary Tables S2-S6. All analyses were performed using SPSS software version 22.0 (IBM Corp., Chicago, IL, USA).

## Results

The anthropometric, metabolic, and instrumental characteristics of the enrolled subjects (*n* = 1,198) are summarized in Table 1. The study cohort was predominantly composed of female subjects (633 vs. 565, *p* < 0.01), who were slightly younger than male subjects (48 ± 13 vs. 49 ± 12 years, *p* = 0.950). The overall population showed a mean BMI of 30.2 ± 6.6 kg/m², with no significant difference between sexes.

**Table 1:** Anthropometric, metabolic and instrumental characteristics of the study groups

Variables	Whole Group	Male	Female	*p*
Sex (n)	1198	565	633	< 0.01
Age (years)	49 (± 13)	49 (± 12)	48 (± 13)	0.950
BMI (Kg/m^2^)	30.2 (± 6.6)	30.1 (± 5.6)	30.2 (± 7.4)	0.982
SBP (mmHg)	132.2(± 15.2)	132.2 (± 15.2)	126.1 (± 18.0)	< 0.0001
DBP (mmHg)	79.9 (± 11.0)	82.2 (± 10.5)	77.9 (± 11.1)	< 0.0001
Tot-Chol (mg/dl)	200.3 (± 39.1)	198.5 (± 38.6)	201.8 (± 39.5)	0.086
HDL-Chol (mg/dl)	51.2 (± 13.7)	45.5 (± 11.3)	56.4 (± 13.6)	< 0.0001
LDL-Chol (mg/dl)	128.5 (± 34.3)	129.3 (± 33.7)	127.9 (± 34.9)	0.639
Triglycerides (mg/dl)	127.9 (± 74.5)	142.9 (± 81.9)	114.7 (± 64.3)	< 0.0001
UA (mg/dl)	5.1 (± 1.4)	5.7 (± 1.3)	4.5 (± 1.2)	< 0.0001
UHR (%)	10.9 (± 4.9)	13.6 (± 5.1)	8.5 (± 3.4)	< 0.0001
FPG (mg/dl)	93.4 (± 10.7)	96.1 (± 10.6)	91.0 (± 10.2)	< 0.0001
FPI (U/l)	13.6(± 8.5)	14.5 (± 9.4)	12.7 (± 7.6)	< 0.0001
HbA1c (%)	5.5 (± 0.4)	5.6 (± 0.3)	5.5 (± 0.3)	0.001
HOMA-IR	3.2 (± 2.1)	3.5 (± 2.3)	2.9 (± 1.8)	< 0.0001
c-IMT (mm)	0.73 (± 0.2)	0.76 (± 0.2)	0.72 (± 0.2)	0.001
LVMI (g/m^2^)	104.1 (± 29.3)	115.0 (± 30.3)	94.3 (± 24.6)	< 0.0001
Hypolipidemic Therapy (%)	18	21	16	0.006
Antihypertensive Therapy (%)	45	48	42	0.029
Smoking habits (Y/N/Ex)	238/678/282	142/228/195	96/450/87	< 0.0001

Data are presented as mean ± SD. Comparisons between the two groups were performed with independent samples t test. Categorical variables were compared by χ2 tests. BMI = body mass index; SBP = systolic blood pressure; DBP = diastolic blood pressure; HDL = high-density lipoprotein; LDL = low-density lipoprotein; UA = uric acid; UHR = uric acid to HDL cholesterol ratio; FPG = fasting plasma glucose; FPI = fasting plasma insulin; HbA1c = glycated hemoglobin; HOMA-IR = homeostasis model assessment index of insulin resistance; c-IMT =carotid intima-media thickness; LVMI = left ventricular mass index

Female subjects exhibited a generally more favorable cardiometabolic profile, characterized by significantly lower SBP and diastolic blood pressure (DBP), triglycerides, UA, UHR, FPG, FPI, HOMA-IR (all *p* < 0.0001) and HbA1c (0.001), as well as higher HDL-Chol levels (*p* < 0.0001). No significant differences were observed between sexes for total and LDL-Chol.

Regarding target organ damage, female subjects showed lower values of both c-IMT and LVMI (*p* = 0.001 and *p* < 0.0001, respectively) and a lower prevalence of type 2 diabetes, drug-treated dyslipidemia, and hypertension.

In the overall population, Pearson correlation analyses (Table 2) demonstrated that UHR was significantly associated with BMI, SBP, DBP, triglycerides, HOMA-IR, HbA1c, FPG, FPI, c-IMT, and LVMI (all *p* < 0.0001). Notably, no significant correlation was observed between UHR and age (*p* = 0.265), Tot-Chol (*p* = 0.058) or LDL-Chol (*p* = 0.131). This pattern suggests that UHR reflects a metabolic risk profile that is partly independent of chronological aging and LDL-driven atherogenic pathways.

**Table 2:** Univariate correlations between UHR and anthropometric and biochemical variables

Variables	Pearson’s correlation coefficient (*r*)	*p*
Age (years)	0.034	0.265
BMI (Kg/m^2^)	0.260	< 0.0001
SBP (mmHg)	0.144	< 0.0001
DBP (mmHg)	0.154	< 0.0001
Tot-Chol (mg/dl)	-0.055	0.058
LDL-Chol (mg/dl)	0.044	0.131
Triglycerides (mg/dl)	0.453	< 0.0001
HbA1c (%)	0.222	< 0.0001
FPG (mg/dl)	0.238	< 0.0001
FPI (U/l)	0.337	< 0.0001
HOMA-IR	0.358	< 0.0001
c-IMT (mm)	0.139	< 0.0001
LVMI (g/m^2^)	0.208	< 0.0001

BMI = body mass index; SBP = systolic blood pressure; DBP = diastolic blood pressure; LDL = low density lipoprotein; HbA1c = glycated hemoglobin; FPG = fasting plasma glucose; FPI = fasting plasma insulin; HOMA-IR = homeostasis model assessment index of insulin resistance; c-IMT = carotid intima-media thickness; LVMI = left ventricular mass index

Of note, the correlation between UHR and LVMI (*r* = 0.208) was numerically stronger than that with c-IMT (*r* = 0.139).

Variables significantly associated with UHR in bivariate analyses (BMI, SBP, DBP, triglycerides, HbA1c, HOMA-IR, FPG and FPI) were subsequently entered into stepwise multivariate linear regression models to identify independent determinants of c-IMT (Table 3) and LVMI (Table 4). Table 3 shows that age emerged as the strongest determinant of c-IMT (β = 0.507, *p* < 0.0001), alongside BMI (β = 0.136, *p* < 0.0001). Importantly, UHR retained a statistically significant, although modest, independent association with c-IMT (β = 0.093 *p* < 0.01) after adjustment for the covariates. In the sensitivity analysis (Model 2), the additional inclusion of lipid-lowering therapy and antihypertensive treatment (including diuretics) did not materially modify these associations, confirming the stability of the model.

**Table 3:** Stepwise multivariable regression analysis of the c-IMT as a dependent variable in the whole population

	Independent Contributors	Standardized Coefficien tβ	*p*
**Model 1**	Age	0.507	< 0.0001
	BMI	0.136	< 0.0001
	**UHR**	**0.093**	**< 0.01**
**Model 2**	Age	0.497	< 0.0001
	BMI	0.153	< 0.0001
	**UHR**	**0.072**	**0.027**
	Hypolipidemic Therapy	0.074	0.025

BMI = body mass index; UHR = uric acid to HDL cholesterol ratio; c-IMT = carotid intima-media thickness

**Table 4:** Stepwise multivariable regression analysis of LVMI as a dependent variable in the whole population

	Independent Contributors	Standardized Coefficient β	*p*
**Model 1**	Age	0.285	< 0.0001
	UHR	0.194	< 0.0001
	SBP	0.156	< 0.0001
	FPG	0.081	0.019
	TG	-0.077	0.030
**Model 2**	Age	0.226	< 0.0001
	**UHR**	**0.172**	**< 0.0001**
	Hypertension Therapy	0.123	< 0.01
	SBP	0.117	0.001
	FPG	0.093	0.01
	FPI	-0.074	< 0.05

SBP = systolic blood pressure; UHR = uric acid to HDL ratio; LVMI = left ventricular mass index

A similar multivariate approach was applied to LVMI (Table 4). In Model 1, age (β = 0.285, *p* < 0.0001), UHR (β = 0.194, *p* < 0.0001), SBP (β = 0.156, *p* < 0.0001), FPG (β = 0.081, *p* = 0.019) and TG (β=-0.077, *p* = 0.030) were independently associated with LVMI. Based on standardized coefficients, UHR showed an independent statistical association with LVMI within this model, comparable to that of SBP, indicating that the metabolic burden captured by UHR may represent a significant, non-negligible contributor to LVMI variability in our cohort. In Model 2, which additionally included lipid-lowering therapy, antihypertensive treatment, the association between UHR and LVMI remained significant (β = 0.172, *p* < 0.0001), suggesting that this relationship was not solely mediated by blood pressure control.

To formally assess sex-specific effects, a General Linear Model was constructed including a sex × UHR interaction term, adjusting for age and SBP. A statistically significant interaction between sex and UHR for LVMI was observed (*p* = 0.024), indicating that the association between UHR and left ventricular mass differs significantly between men and women. Consequently, further analyses were stratified by sex. In women (Table 5), UHR was independently associated with LVMI in multivariate Model 1 (β = 0.114, *p* < 0.01). This association persisted after further adjustment for antihypertensive or hypolipidemic treatment, smoking habit, and menopausal status (β = 0.125, *p* < 0.01). In contrast, stepwise multivariate regression analyses performed in male subjects did not reveal any significant association between UHR and LVMI or other examined covariates (Supplementary Table S5).

**Table 5:** Stepwise multivariable regression analysis of LVMI as a dependent variable in female population

	Independent Contributors	Standardized Coefficient β	*p*
**Model 1**	Age	0.292	< 0.0001
	SBP	0.213	< 0.0001
	**UHR**	**0.114**	**< 0.01**
**Model 2**	Age	0.306	< 0.0001
	DBP	0.176	< 0.0001
	Hypertension Therapy Smoking habit	0.144 -0.099	0.012 0.033
	**UHR**	**0.125**	**< 0.01**

SBP = systolic blood pressure; UHR = uric acid to HDL ratio; LVMI = left ventricular mass index

**Fig. 1:**
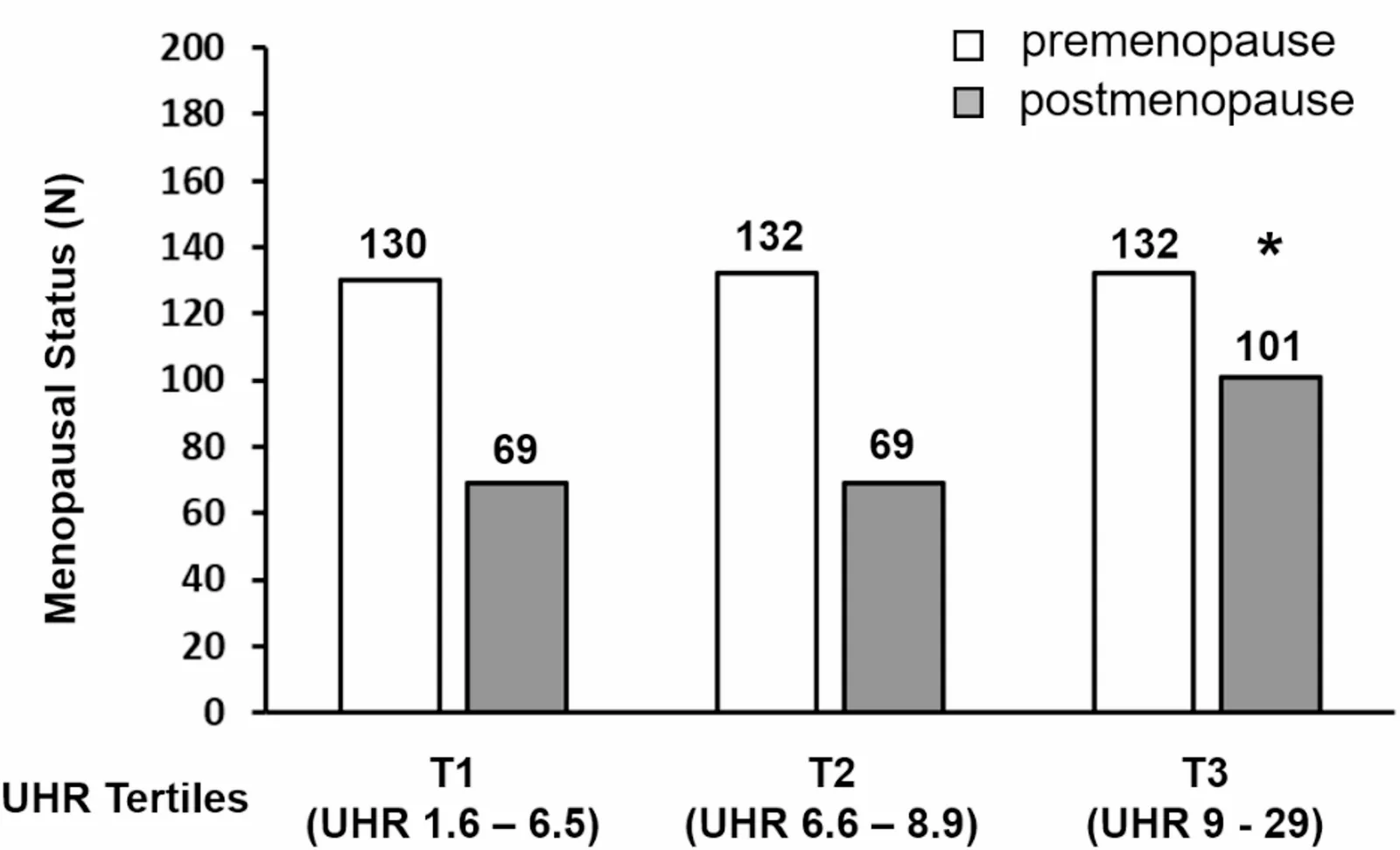
Menopause status in the female study population, stratified by UHR (Uric Acid-to-HDL-Cholesterol Ratio) tertiles. Tertiles are defined based on population distribution: T1 (UHR 1.6–6.5), T2 (UHR 6.6–8.9), and T3 (UHR 9–29). *p = 0.03 for Postmenopause prevalence in T3 versus T1 and T2

Among the female population, 43% were postmenopausal and 57% were of reproductive age. Mean UHR was significantly higher in postmenopausal compared with premenopausal women (8.9 ± 3.2 vs. 8.2 ± 3.7, *p* < 0.01). When women were stratified into UHR tertiles, a progressive increase in the proportion of postmenopausal subjects was observed across tertiles, with a significant linear trend (Fig. 1, *p* = 0.03).

Finally, multivariate analyses stratified by menopausal status in women are presented in Table 6. After adjustment for potential confounders including age, BMI, SBP, DBP, TG, HbA1c, HOMA-IR, FPG, FPI, UHR was not significantly associated with LVMI in premenopausal women. In contrast, in postmenopausal women, UHR remained an independent determinant of LVMI (β = 0.164, *p* = 0.033).

**Table 6:** Stepwise multivariable regression analysis of the LVMI as a dependent variable in menopausal female group

Independent Contributors	Standardized Coefficient β	p
SBP	0.275	<0.0001
**UHR**	**0.164**	**0.033**

SBP = systolic blood pressure; UHR = uric acid to HDL cholesterol ratio; LVMI = left ventricular mass index

## Discussion

In this large, well-characterized cohort, we demonstrate that UHR is independently associated with subclinical cardiovascular organ damage, as assessed by c-IMT and LVMI. A key finding of the present study is that this association is more consistent and quantitatively stronger for LVMI than for c-IMT, highlighting myocardial remodeling as the principal phenotype linked to UHR. Furthermore, we observed a clear sex-related heterogeneity in this relationship: while UHR was linked to LVMI in the overall population, this association was largely prominent in women and was supported by a significant sex × UHR interaction in fully adjusted models (*p* = 0.024). Stratified analyses confirmed that UHR was associated with LVMI in women, particularly those in postmenopausal status, whereas no significant association was detected in men.

Consistently, postmenopausal women exhibited significantly higher mean UHR values than premenopausal women (8.9 ± 3.2 vs. 8.2 ± 3.7, *p* < 0.01), and the proportion of postmenopausal women increased across UHR tertiles, with a significant linear-by-linear association (*p* = 0.03), supporting a graded relationship between UHR and menopausal status. These findings indicate that UHR captures a cardiometabolic burden that is differentially linked to myocardial remodeling according to sex and hormonal status.

Left ventricular hypertrophy, defined as an inappropriate increase in LVM beyond compensatory hemodynamic needs, is a major determinant of CV and all-cause morbidity and mortality [3]. While elevated blood pressure is the primary driver of left ventricular remodeling, metabolic and neurohormonal factors substantially contribute to cardiac structural changes [4, 5]. An independent relationship between insulin levels, insulin resistance and LVM is well-established [39–41]; high levels of insulinemia are associated with reduced levels of insulin-like growth factor-1 and increased action of the pituitary growth hormone with known effects on myocardial trophism, proliferation and water retention [42]. In addition, insulin resistance is associated with subclinical inflammation [43], increased sympathetic stimulation and peripheral vascular resistance [44, 45], as well as the modulation of vasoactive substances and activation of the renin-angiotensin-aldosterone system (RAAS) [46]. Prolonged exposure to inappropriately elevated aldosterone levels may induce cardiac inflammation and interstitial fibrosis, resulting in maladaptive cardiac remodeling and alterations in left ventricle structure and function [47]. Regarding the molecular mechanisms involved, recent studies have proposed the involvement of aldosterone as a trigger for the activation of p38 mitogen-activated protein kinase (MAPK) and nicotinamide riboside kinase 1/2 (nRK1/2), which have been implicated in signal transduction pathways associated with cardiac hypertrophy [48].

Sundström et al. demonstrated that dyslipidemia and an unfavorable fatty acid profile are risk indicators for developing LVH to a similar extent as hypertension and obesity [49]; lipid accumulation in cardiomyocytes or in the interstitial space, local and systemic inflammation, insulin resistance, oxidative stress, autonomic nervous system dysfunction and RAAS activation are among the main proposed mechanisms [47, 50, 51]. Adipose tissue stimulates aldosterone secretion from the adrenal gland, while very low-density lipoproteins (VLDL) may induce aldosterone overproduction through the induction of steroidogenic acute regulatory protein (StAR) and aldosterone synthase (CYP11B2) expression [52].

Our study population consisted of 1198 individuals, with an average BMI of 30.2 ± 6.6, increased markers of insulin resistance and elevated LDL-Chol levels, all well known risk factors associated with LVMI and c-IMT.

Elevated UA has been hypothesized to promote myocardial remodeling through increased xanthine oxidase (XO) activity, which is thought to enhance reactive oxygen species production within cardiomyocytes and the cardiac interstitium [53]. Experimental studies have shown that XO-derived oxidative stress can impair mitochondrial function, activate pro-inflammatory signaling pathways, and stimulate fibroblast proliferation, ultimately promoting interstitial fibrosis and cardiomyocyte hypertrophy, key determinants of increased LVMI [54, 55]. In parallel, elevated UA has the potential to impair HDL functionality. Inflammatory and oxidative environments may modify apolipoprotein A-1, reduce paraoxonase-1 activity, and potentially convert HDL from an anti-oxidant to a pro-oxidant particle. Although not directly assessed in our study, such dysfunctional HDL would be less effective in reverse cholesterol transport, endothelial protection, and suppression of vascular inflammation, thereby amplifying myocardial and vascular injury. The combination of increased UA and impaired HDL function, captured by UHR, provides a biologically coherent explanation for its association with LVMI [56] that requires direct confirmation in future mechanistic studies. In this context, the independent association between UHR and LVMI observed in our study is consistent with the hypothesis that composite metabolic markers may reflect myocardial stress beyond traditional hemodynamic determinants.

The sex-related differences observed in the association between UHR and LVMI are biologically plausible. Estrogens exert uricosuric, anti-inflammatory, and vasoprotective effects, which may attenuate the adverse CV impact of UA and preserve HDL functionality in premenopausal women [57]. After menopause, the loss of estrogen-mediated protection is accompanied by unfavorable changes in both UA metabolism and HDL-Chol levels, potentially enhancing the pathological interaction hypothesized to be captured by UHR. This is consistent with previous evidence indicating that the association between UA and LVH is more evident in postmenopausal women than in premenopausal women [58]. Importantly, our observation that postmenopausal women had higher UHR levels, together with the increasing proportion of postmenopausal subjects across UHR tertiles, strengthens the concept that menopause is associated with a metabolic milieu more closely linked to myocardial remodeling. Despite lower absolute UHR values in women compared with men, we observed a significant and independent association between UHR and LVMI in postmenopausal women, potentially suggesting increased myocardial susceptibility to this combined metabolic signal after menopause. This potentially links the loss of hormonal protection to the “unmasking” of a latent metabolic vulnerability in the aging female heart.

With regard to vascular damage, UHR was independently associated with c-IMT in the overall population, in line with previous evidence linking this index to early atherosclerotic remodeling and arterial wall thickening [7, 59]. However, unlike LVMI, the association between UHR and c-IMT did not demonstrate a consistent sex-specific pattern, suggesting that myocardial remodeling may be more sensitive than carotid wall thickening to the biological context underlying UHR. Furthermore, while c-IMT was strongly dependent on age, UHR was not, indicating that UHR likely reflects a functional metabolic risk rather than structural vascular aging per se.

An additional relevant aspect of our findings is that both UA and HDL-Chol levels were largely within conventional reference ranges in the study population. Nevertheless, their ratio identified individuals, particularly postmenopausal women, with evidence of subclinical cardiac damage. This observation supports the hypothesis that UHR conceptually integrates two biologically interrelated pathways and may capture CV risk not apparent when UA or HDL-Chol are considered in isolation [60].

Our findings extend those of Yu et al. [58], who reported in 2015 an association between serum UA and LVH that was more pronounced in postmenopausal women. Unlike previous studies focusing solely on single-biomarker pools, our results suggest that UHR, by reflecting the crosstalk between oxidative burden and proactive lipid transport, is consistently associated with subclinical structural damage. This line of clinical evidence is strongly supported by recent large-scale epidemiological data from the NHANES cohort, which identified UHR as a highly sensitive metabolic indicator specifically linked to increased obesity metrics and abdominal adiposity in postmenopausal women [31]. By bridging these observations, our study expands the clinical paradigm, suggesting that the postmenopausal increase in UHR translates beyond general adiposity into advanced subclinical cardiovascular alterations, namely myocardial remodelling.

From a clinical perspective, the translation of these findings into routine practice deserves careful consideration. While population-wide imaging screening for subclinical cardiovascular organ damage remains unfeasible due to cost and time constraints, the clinical selection of high-risk individuals who warrant deeper diagnostic evaluation is heavily required. In this context, the UHR index emerges as an inexpensive and easily interpretable tool that could effectively support riskstratification in primary care. Rather than serving as a standalone predictive or diagnostic screening tool, an elevated UHR could act as an accessible biochemical indicator, particularly in postmenopausal women, to identify silent metabolic vulnerabilities and guide clinicians toward prioritized cardiovascular workups (such as echocardiography or carotid ultrasound). Nonetheless, prospective and multicentre studies remain essential to establish clinically meaningful UHR cutoff values and to evaluate its formal incremental utility through modern reclassification metrics.

Several limitations should be acknowledged: the cross-sectional design limits causal inference and temporal interpretation; we lacked longitudinal follow-up to assess whether UHR predicts progression of LVMI or incident cardiovascular events; we did not directly measure oxidative stress markers or HDL functionality, precluding mechanistic confirmation; potential residual confounding cannot be excluded despite multivariable adjustment. Additionally, menopausal status was defined according to the STRAW + 10 criteria based on clinical history [34]. While these criteria are robust for clinical staging, the lack of FSH levels may represent a limitation in identifying women in the late perimenopausal phase. Finally, the study population was limited to Caucasian subjects, which may limit generalizability to other ethnic groups, as well as to lower-risk general populations. However, the trans-ethnic applicability of UHR in the postmenopausal setting has been recently validated in a large, multi-ethnic, non-European cohort from the NHANES database [31]. While their findings corroborate the relevance of UHR as a metabolic indicator across diverse populations, future prospective, international, and multicenter studies remain essential to externally validate our findings across diverse risk profiles and to facilitate translation into clinical practice.

In conclusion, UHR is independently associated with subclinical CV organ damage and shows a clear sex-specific relationship with LVM, particularly in postmenopausal women. These findings suggest that UHR may serve as a simple, widely available biomarker that refines early CV risk stratification in women after menopause. However, prospective studies are warranted to determine whether UHR predicts progression of LVMI and incident CV events, and whether its inclusion improves risk prediction beyond established clinical models.

## Supplementary Information

Supplementary Material 1

## Data Availability

The authors declare that all data supporting the findings of this study are available from the corresponding author on reasonable request.

## References

[1] Mensah GA, Roth GA, Fuster V. The Global Burden of Cardiovascular Diseases and Risk Factors: 2020 and Beyond. J Am Coll Cardiol. 2019;74:2529–32. 10.1016/j.jacc.2019.10.009. 31727292

[2] Levy D, Labib SB, Anderson KM, et al. Determinants of sensitivity and specificity of electrocardiographic criteria for left ventricular hypertrophy. Circulation. 1990;81:815–20. 10.1161/01.cir.81.3.815. 2137733

[3] Yildiz M, Oktay AA, Stewart MH, et al. Left ventricular hypertrophy and hypertension. Prog Cardiovasc Dis. 2020;63:10–21. 10.1016/j.pcad.2019.11.009. 31759953

[4] Saba PS, Roman MJ, Pini R, et al. Relation of arterial pressure waveform to left ventricular and carotid anatomy in normotensive subjects. J Am Coll Cardiol. 1993;22:1873–80. 10.1016/0735-1097(93)90772-s. 8245342

[5] de Simone G, Palmieri V, Bella JN, et al. Association of left ventricular hypertrophy with metabolic risk factors: the HyperGEN study. J Hypertens. 2002;20:323–31. 10.1097/00004872-200202000-00024. 11821719

[6] Zieman SJ, Melenovsky V, Kass DA. Mechanisms, pathophysiology, and therapy of arterial stiffness. Arterioscler Thromb Vasc Biol. 2005;25:932–43. 10.1161/01.ATV.0000160548.78317.29. 15731494

[7] Carpenter M, Sinclair H, Kunadian V. Carotid Intima Media Thickness and Its Utility as a Predictor of Cardiovascular Disease: A Review of Evidence. Cardiol Rev. 2016;24:70–5. 10.1097/CRD.0000000000000077. 26825762

[8] Tassone EJ, Cimellaro A, Perticone M, et al. Uric Acid Impairs Insulin Signaling by Promoting Enpp1 Binding to Insulin Receptor in Human Umbilical Vein Endothelial Cells. Front Endocrinol. 2018;9:98. 10.3389/fendo.2018.00098. PMC5879120 29619007

[9] Fiorentino TV, Sesti F, Succurro E, et al. Higher serum levels of uric acid are associated with a reduced insulin clearance in non-diabetic individuals. Acta Diabetol. 2018;55:835–42. 10.1007/s00592-018-1153-8. 29774469

[10] Gaubert M, Bardin T, Cohen-Solal A, et al. Hyperuricemia and Hypertension, Coronary Artery Disease, Kidney Disease: From Concept to Practice. Int J Mol Sci. 2020;21:4066. 10.3390/ijms21114066. PMC7312288 33561034

[11] Katsiki N, Dimitriadis GD, Mikhailidis DP. Serum Uric Acid and Diabetes: From Pathophysiology to Cardiovascular Disease. Curr Pharm Des. 2021;27:1941–51. 10.2174/1381612827666210104124320. 33397230

[12] Perticone M, Maio R, Shehaj E, et al. Sex-related differences for uric acid in the prediction of cardiovascular events in essential hypertension. A population prospective study. Cardiovasc Diabetol. 2023;22:298. 10.1186/s12933-023-02006-z. PMC10621159 37915077

[13] Perticone M, Tripepi G, Maio R, et al. Risk reclassification ability of uric acid for cardiovascular outcomes in essential hypertension. Int J Cardiol. 2017;243:473–8. 10.1016/j.ijcard.2017.05.051. 28528984

[14] Ndrepepa G. Uric acid and cardiovascular disease. Clin Chim Acta Int J Clin Chem. 2018;484:150–63. 10.1016/j.cca.2018.05.046. 29803897

[15] Maruhashi T, Hisatome I, Kihara Y, et al. Hyperuricemia and endothelial function: From molecular background to clinical perspectives. Atherosclerosis. 2018;278:226–31. 10.1016/j.atherosclerosis.2018.10.007. 30326405

[16] Yu W, Cheng J-D. Uric Acid and Cardiovascular Disease: An Update From Molecular Mechanism to Clinical Perspective. Front Pharmacol. 2020;11:582680. 10.3389/fphar.2020.582680. PMC7701250 33304270

[17] Kuo C-F, Yu K-H, Luo S-F, et al. Role of uric acid in the link between arterial stiffness and cardiac hypertrophy: a cross-sectional study. Rheumatology. 2010;49:1189–96. 10.1093/rheumatology/keq095. 20371503

[18] Kim SY, Guevara JP, Kim KM, et al. Hyperuricemia and coronary heart disease: a systematic review and meta-analysis. Arthritis Care Res. 2010;62:170–80. 10.1002/acr.20065. PMC3156692 20191515

[19] Zhang W, Iso H, Murakami Y, et al. Serum Uric Acid and Mortality Form Cardiovascular Disease: EPOCH-JAPAN Study. J Atheroscler Thromb. 2016;23:1365–6. 10.5551/jat.Er31591. PMC5221499 27904050

[20] Kamei K, Konta T, Hirayama A, et al. Associations between serum uric acid levels and the incidence of nonfatal stroke: a nationwide community-based cohort study. Clin Exp Nephrol. 2017;21:497–503. 10.1007/s10157-016-1311-7. 27405620

[21] von Eckardstein A, Nordestgaard BG, Remaley AT, et al. High-density lipoprotein revisited: biological functions and clinical relevance. Eur Heart J. 2023;44:1394–407. 10.1093/eurheartj/ehac605. PMC10119031 36337032

[22] Barter P, Gotto AM, LaRosa JC, et al. HDL cholesterol, very low levels of LDL cholesterol, and cardiovascular events. N Engl J Med. 2007;357:1301–10. 10.1056/NEJMoa064278. 17898099

[23] Zhong G-C, Huang S-Q, Peng Y, et al. HDL-C is associated with mortality from all causes, cardiovascular disease and cancer in a J-shaped dose-response fashion: a pooled analysis of 37 prospective cohort studies. Eur J Prev Cardiol. 2020;27:1187–203. 10.1177/2047487320914756. 32283957

[24] Vekic J, Jelic-Ivanovic Z, Spasojevic-Kalimanovska V, et al. High serum uric acid and low-grade inflammation are associated with smaller LDL and HDL particles. Atherosclerosis. 2009;203:236–42. 10.1016/j.atherosclerosis.2008.05.047. 18603253

[25] Hu X, Liu J, Li W, et al. Elevated serum uric acid was associated with pre-inflammatory state and impacted the role of HDL-C on carotid atherosclerosis. Nutr Metab Cardiovasc Dis NMCD. 2022;32:1661–9. 10.1016/j.numecd.2022.03.026. 35469728

[26] Aktas G, Khalid A, Kurtkulagi O, et al. Poorly controlled hypertension is associated with elevated serum uric acid to HDL-cholesterol ratio: a cross-sectional cohort study. Postgrad Med. 2022;134:297–302. 10.1080/00325481.2022.2039007. 35142235

[27] Yu X, Sun F, Ming J, et al. Serum uric acid to high-density lipoprotein cholesterol ratio is a promising marker for identifying metabolic syndrome in nondiabetic Chinese men. Postgrad Med. 2023;135:741–9. 10.1080/00325481.2023.2263372. 37750609

[28] Liu P, Li J, Yang L, et al. Association between cumulative uric acid to high-density lipoprotein cholesterol ratio and the incidence and progression of chronic kidney disease. Front Endocrinol. 2023;14:1269580. 10.3389/fendo.2023.1269580. PMC10753577 38155948

[29] Zhou X, Xu J. Association between serum uric acid-to-high-density lipoprotein cholesterol ratio and insulin resistance in an American population: A population-based analysis. J Diabetes Investig. 2024;15:762–71. 10.1111/jdi.14170. PMC11143423 38407574

[30] Rubino M, Massimino M, Mancuso E, et al. Serum uric acid to HDL-Chol ratio (UHR) is associated with insulin resistance/sensitivity in individuals without diabetes. Acta Diabetol. 2026;63:87–95. 10.1007/s00592-025-02576-2. PMC12847180 40864241

[31] Tian J, Huang Y, Xiao Z, et al. The association between the uric acid to high-density lipoprotein cholesterol ratio (UHR) index and obesity in postmenopausal women: a cross-sectional analysis based on the NHANES. Lipids Health Dis. 2025;24:277. 10.1186/s12944-025-02715-2. PMC12412247 40913235

[32] Andreozzi F, Succurro E, Mancuso MR, et al. Metabolic and cardiovascular risk factors in subjects with impaired fasting glucose: the 100 versus 110 mg/dL threshold. Diabetes Metab Res Rev. 2007;23:547–50. 10.1002/dmrr.724. 17311284

[33] Andreozzi F, Mannino GC, Perticone M, et al. Elevated 1-h post-load plasma glucose levels in subjects with normal glucose tolerance are associated with a pro-atherogenic lipid profile. Atherosclerosis. 2017;256:15–20. 10.1016/j.atherosclerosis.2016.11.020. 27940375

[34] Harlow SD, Gass M, Hall JE, et al. Executive summary of the Stages of Reproductive Aging Workshop + 10: addressing the unfinished agenda of staging reproductive aging. Menopause. 2012;19:387–95. 10.1097/gme.0b013e31824d8f40. PMC3340903 22343510

[35] Casella IB, Presti C, Porta RMP, et al. A practical protocol to measure common carotid artery intima-media thickness. Clinics. 2008;63:515–20. 10.1590/s1807-59322008000400017. PMC2664129 18719764

[36] Devereux RB, Alonso DR, Lutas EM, et al. Echocardiographic assessment of left ventricular hypertrophy: comparison to necropsy findings. Am J Cardiol. 1986;57:450–8. 10.1016/0002-9149(86)90771-x. 2936235

[37] Matthews DR, Hosker JP, Rudenski AS, et al. Homeostasis model assessment: insulin resistance and beta-cell function from fasting plasma glucose and insulin concentrations in man. Diabetologia. 1985;28:412–9. 10.1007/BF00280883. 3899825

[38] Du Bois D, Du Bois EF. A formula to estimate the approximate surface area if height and weight be known. 1916. Nutrition. 1989;5:303–11. discussion 312–313. 2520314

[39] Sundström J, Lind L, Nyström N, et al. Left ventricular concentric remodeling rather than left ventricular hypertrophy is related to the insulin resistance syndrome in elderly men. Circulation. 2000;101:2595–600. 10.1161/01.cir.101.22.2595. 10840010

[40] Galvan AQ, Galetta F, Natali A, et al. Insulin resistance and hyperinsulinemia: No independent relation to left ventricular mass in humans. Circulation. 2000;102:2233–8. 10.1161/01.cir.102.18.2233. 11056098

[41] Cauwenberghs N, Knez J, Thijs L, et al. Relation of Insulin Resistance to Longitudinal Changes in Left Ventricular Structure and Function in a General Population. J Am Heart Assoc. 2018;7:e008315. 10.1161/JAHA.117.008315. PMC5907600 29574459

[42] Sesti G, Sciacqua A, Scozzafava A, et al. Effects of growth hormone and insulin-like growth factor-1 on cardiac hypertrophy of hypertensive patients. J Hypertens. 2007;25:471–7. 10.1097/HJH.0b013e3280112b63. 17211256

[43] Shah RV, Abbasi SA, Heydari B, et al. Insulin resistance, subclinical left ventricular remodeling, and the obesity paradox: MESA (Multi-Ethnic Study of Atherosclerosis). J Am Coll Cardiol. 2013;61:1698–706. 10.1016/j.jacc.2013.01.053. PMC4114341 23500236

[44] Landsberg L. Pathophysiology of obesity-related hypertension: role of insulin and the sympathetic nervous system. J Cardiovasc Pharmacol. 1994;23(Suppl 1):S1–8. 7519690

[45] Takagi M, Tanaka Y, Yamasaki Y, et al. Responsiveness of insulin-induced cardiac sympathetic nerve activation associates with blood pressure regulation in diabetics. Am J Physiol Endocrinol Metab. 2003;284:E1022–1026. 10.1152/ajpendo.00169.2002. 12569084

[46] Iacobellis G, Ribaudo MC, Zappaterreno A, et al. Relationship of insulin sensitivity and left ventricular mass in uncomplicated obesity. Obes Res. 2003;11:518–24. 10.1038/oby.2003.73. 12690080

[47] Catena C, Colussi G, Brosolo G, et al. Aldosterone and Left Ventricular Remodeling. Horm Metab Res Horm Stoffwechselforschung Horm Metab. 2015;47:981–6. 10.1055/s-0035-1565055. 26566103

[48] Weber KT, Brilla CG. Pathological hypertrophy and cardiac interstitium. Fibrosis and renin-angiotensin-aldosterone system. Circulation. 1991;83:1849–65. 10.1161/01.cir.83.6.1849. 1828192

[49] Sundström J, Lind L, Vessby B, et al. Dyslipidemia and an unfavorable fatty acid profile predict left ventricular hypertrophy 20 years later. Circulation. 2001;103:836–41. 10.1161/01.cir.103.6.836. 11171792

[50] Arnlöv J, Lind L, Sundström J, et al. Insulin resistance, dietary fat intake and blood pressure predict left ventricular diastolic function 20 years later. Nutr Metab Cardiovasc Dis NMCD. 2005;15:242–9. 10.1016/j.numecd.2004.10.002. 16054547

[51] Piratello AC, Moraes-Silva I, Paulini J, et al. Renin angiotensin system and cardiac hypertrophy after sinoaortic denervation in rats. Clinics. 2010;65:1345–50. 10.1590/s1807-59322010001200019. PMC3020347 21340225

[52] Tsai Y-Y, Rainey WE, Bollag WB. Very low-density lipoprotein (VLDL)-induced signals mediating aldosterone production. J Endocrinol. 2017;232:R115–29. 10.1530/JOE-16-0237. PMC8310676 27913572

[53] Alem MM, Aldosari SR, Alkahmous AA, et al. Effect of Long-Term Allopurinol Therapy on Left Ventricular Mass Index in Patients with Ischemic Heart Disease; A Cross-Sectional Study. Vasc Health Risk Manag. 2019;15:539–50. 10.2147/VHRM.S226009. PMC6903809 31827327

[54] Minhas KM, Saraiva RM, Schuleri KH, et al. Xanthine oxidoreductase inhibition causes reverse remodeling in rats with dilated cardiomyopathy. Circ Res. 2006;98:271–9. 10.1161/01.RES.0000200181.59551.71. 16357304

[55] George J, Struthers AD. The role of urate and xanthine oxidase inhibitors in cardiovascular disease. Cardiovasc Ther. 2008;26:59–64. 10.1111/j.1527-3466.2007.00029.x. 18466421

[56] Navab M, Reddy ST, Van Lenten BJ, et al. HDL and cardiovascular disease: atherogenic and atheroprotective mechanisms. Nat Rev Cardiol. 2011;8:222–32. 10.1038/nrcardio.2010.222. 21304474

[57] Hak AE, Choi HK. Menopause, postmenopausal hormone use and serum uric acid levels in US women–the Third National Health and Nutrition Examination Survey. Arthritis Res Ther. 2008;10:R116. 10.1186/ar2519. PMC2592803 18822120

[58] Yu S, Yang H, Guo X, et al. Hyperuricemia is independently associated with left ventricular hypertrophy in post-menopausal women but not in pre-menopausal women in rural Northeast China. Gynecol Endocrinol Off J Int Soc Gynecol Endocrinol. 2015;31:736–41. 10.3109/09513590.2015.1056730. 26376959

[59] O’Leary DH, Bots ML. Imaging of atherosclerosis: carotid intima-media thickness. Eur Heart J. 2010;31:1682–9. 10.1093/eurheartj/ehq185. 20542989

[60] Li Z, Liu Q, Yao Z. The serum uric acid-to-high-density lipoprotein cholesterol ratio is a predictor for all-cause and cardiovascular disease mortality: a cross-sectional study. Front Endocrinol. 2024;15:1417485. 10.3389/fendo.2024.1417485. PMC11427315 39345882

