# Supplementary material for: Sex differences in the association of uric acid to HDL-cholesterol ratio (UHR) with subclinical cardiovascular damage
Source: Biol Sex Differ. 2026 Jul 22;17:168. doi: 10.1186/s13293-026-00956-z (PMC13679248; doi:10.1186/s13293-026-00956-z)
Supplement: Supplementary file 1 [file 13293_2026_956_MOESM1_ESM.docx]

| **Table S1. Baseline Pharmacological Treatments of the Study Population Stratified by Sex and Menopausal Status.** | | | | |  |
| --- | --- | --- | --- | --- | --- |
| **Medication Class** | **Whole Group (N=1198)** | **Males (n=565)** | **Females (n=633)** | **Menopause (n=239)** |  |
|  | **n (%)** | **n (% of males)** | **n (% of females)** | **n (% of menopausal)** |  |
| **Antihypertensive agents** |  |  |  |  |  |
| - RAAS Inhibitors (ACEi/ARBs) | 410 (34.2%) | 216 (38.2%) | 194 (30.6%) | 116 (48.5%) |  |
| - Diuretics | 193 (16.1%) | 92 (16.3%) | 101 (16.0%) | 61 (25.5%) |  |
| - Beta-blockers | 173 (14.4%) | 81 (14.3%) | 92 (14.5%) | 53 (22.2%) |  |
| - Calcium Channel Blockers | 148 (12.4%) | 87 (15.4%) | 61 (9.6%) | 31 (13.0%) |  |
| - Others (alpha-bl./vasod.) | 27 (2.3%) | 20 (3.5%) | 7 (1.1%) | 5 (2.1%) |  |
| **Lipid & Antithrombotic** |  |  |  |  |  |
| - Statins | 127 (10.6%) | 80 (14.2%) | 47 (7.4%) | 38 (15.9%) |  |
| - Fibrates | 4 (0.3%) | 4 (0.7%) | 0 (0.0%) | 0 (0.0%) |  |
| - Antiaggregants/Anticoag. | 94 (7.8%) | 55 (9.7%) | 39 (6.2%) | 35 (14.6%) |  |
| **Glucose-lowering** |  |  |  |  |  |
| - Metformin | 9 (0.8%) | 2 (0.4%) | 7 (1.1%) | 3 (1.3%) |  |
| - Other treatments/Insulin | 3 (0.3%) | 1 (0.2%) | 2 (0.3%) | 2 (0.8%) |  |
| **Hormonal Therapies** |  |  |  |  |  |
| - Thyroid Therapy | 29 (2.4%) | 5 (0.9%) | 24 (3.8%) | 10 (4.2%) |  |
| - Estroprogestins | 0 (0.0%) | 0 (0.0%) | 0 (0.0%) | 0 (0.0%) |  |
| - Corticosteroids | 2 (0.2%) | 1 (0.2%) | 1 (0.2%) | 1 (0.4%) |  |
| **Other Medications** | 43 (3.6%) | 14 (2.5%) | 29 (4.6%) | 11 (4.6%) |  |
| Data are presented as absolute numbers and percentages, n (%). For the subgroups, percentages are calculated relative to the total number of subjects within each specific column. The "Menopause" column includes only postmenopausal women dynamically classified according to the STRAW+10 criteria. Abbreviations*:* RAAS, Renin-Angiotensin-Aldosterone System; ACEi, Angiotensin-Converting Enzyme Inhibitors; ARBs, Angiotensin II Receptor Blockers; alpha-bl., alpha-blockers; vasod., vasodilators; Antiaggregants/Anticoag., Antiplatelet and Anticoagulant agents. | | | | |  |
|  |  |  |  |  |  |
|  |  |  |  |  |  |
|  |  |  |  |  |  |
|  |  |  |  |  |  |

Full step-by-step model building and diagnostic statistics were provided to the reviewers during the peer-review process and are available upon request.

| **Table S2. Final Multivariate Regression Models for cIMT (Total Population)** | | | |  |
| --- | --- | --- | --- | --- |
| **Predictors** | **Standardized β** | **p-value** | **VIF** |  |
| **MODEL 1: Clinical Model (Adjusted R² = 0.277)** | | | |  |
| Age | 0.507 | <.001 | 1.10 |  |
| BMI | 0.136 | <.001 | 1.10 |  |
| **UHR** | **0.093** | **0.003** | **1.10** |  |
| **MODEL 2: Sensitivity Model (Adjusted R² = 0.291)** | | | |  |
| Age | 0.497 | <.001 | 1.11 |  |
| BMI | 0.153 | <.001 | 1.11 |  |
| Lipid-lowering Therapy | 0.074 | 0.025 | 1.12 |  |
| **UHR** | **0.072** | **0.027** | **1.11** |  |
| Model 1: Primary clinical model using stepwise selection. Variables excluded (p > 0.05): SBP, DBP, HbA1c, FPG, FPI, Triglycerides, Smoking habit, Antihypertensive and Lipid-lowering therapies. Model 2: Sensitivity model adjusting for pharmacological treatments. Variables excluded (p > 0.05): SBP, DBP, HbA1c, FPG, FPI, Triglycerides, Smoking habit and Antihypertensive therapy. Abbreviations: cIMT, carotid Intima-Media Thickness; UHR, Uric Acid to HDL-cholesterol Ratio; BMI, Body Mass Index; VIF, Variance Inflation Factor. | | | |  |
|  |  |  |  |  |
|  |  |  |  |  |
|  |  |  |  |  |
|  |  |  |  |  |
|  |  |  |  |  |
|  |  |  |  |  |
|  |  |  |  |  |

Full step-by-step model building and diagnostic statistics were provided to the reviewers during the peer-review process and are available upon request.

| **Table S3. Final Multivariate Regression Models for LVMI (Total Population)** | | | |  |
| --- | --- | --- | --- | --- |
| **Predictors** | **Standardized β** | **p-value** | **VIF** |  |
| **MODEL 1: Clinical Model (Adjusted R² = 0.197)** | | | |  |
| Age | 0.285 | <.001 | 1.24 |  |
| **UHR** | **0.194** | **<.001** | **1.24** |  |
| SBP | 0.156 | <.001 | 1.16 |  |
| FPG (Glucose) | 0.081 | 0.019 | 1.23 |  |
| Triglycerides | -0.077 | 0.030 | 1.30 |  |
| **MODEL 2: Sensitivity Model (Adjusted R² = 0.210)** | | | |  |
| Age | 0.226 | <.001 | 1.34 |  |
| **UHR** | **0.172** | **<.001** | **1.07** |  |
| Antihypertensive Therapy | 0.123 | 0.002 | 1.45 |  |
| SBP | 0.117 | 0.001 | 1.15 |  |
| FPG (Glucose) | 0.093 | 0.011 | 1.24 |  |
| FPI (Insulin) | -0.074 | 0.041 | 1.21 |  |
| Model 1: Primary clinical model using stepwise selection. Variables excluded (p > 0.05): BMI, DBP, HbA1c, Smoking habit, FPI, Antihypertensive and Lipid-lowering therapies. Model 2: Sensitivity model adjusting for pharmacological treatments. Variables excluded (p > 0.05): BMI, DBP, HbA1c, Triglycerides, Smoking habit and Lipid-lowering therapy. Abbreviations: LVMI, Left Ventricular Mass Index; UHR, Uric Acid to HDL-cholesterol Ratio; SBP, Systolic Blood Pressure; FPG, Fasting Plasma Glucose; FPI, Fasting Plasma Insulin; VIF, Variance Inflation Factor. | | | |  |
|  |  |  |  |  |
|  |  |  |  |  |
|  |  |  |  |  |
|  |  |  |  |  |

Full step-by-step model building and diagnostic statistics were provided to the reviewers during the peer-review process and are available upon request.

| **Table S4. Final Multivariate Regression Models for LVMI (Females Only, n=633)** | | | |  |
| --- | --- | --- | --- | --- |
| **Predictors** | **Standardized β** | **p-value** | **VIF** |  |
| **MODEL 1: Clinical Model (Adjusted R² = 0.199)** | | | |  |
| Age | 0.292 | <.001 | 1.01 |  |
| SBP | 0.213 | <.001 | 1.01 |  |
| **UHR** | **0.114** | **0.007** | **1.02** |  |
| **MODEL 2: Sensitivity Model (Adjusted R² = 0.250)** | | | |  |
|  |  |  |  |  |
| Age | 0.306 | <.001 | 1.55 |  |
| DBP | 0.176 | <.001 | 1.56 |  |
| Antihypertensive Therapy | 0.144 | 0.012 | 1.18 |  |
| **UHR** | **0.125** | **0.008** | **1.02** |  |
| Smoking Habit | -0.099 | 0.033 | 1.02 |  |
| Model 1: Primary clinical model using stepwise selection. Variables excluded (p > 0.05): BMI, DBP, HbA1c, FPG, FPI, Triglycerides, Smoking habit, Menopausal status, Antihypertensive and Lipid-lowering therapies. Model 2: Sensitivity model adjusting for pharmacological treatments. Variables excluded (p > 0.05): BMI, SBP, HbA1c, FPG, FPI, Triglycerides, Menopausal status and Lipid-lowering therapy. Abbreviations: LVMI, Left Ventricular Mass Index; UHR, Uric Acid to HDL-cholesterol Ratio; SBP, Systolic Blood Pressure; DBP, Diastolic Blood Pressure; VIF, Variance Inflation Factor. | | | |  |
|  |  |  |  |  |
|  |  |  |  |  |
|  |  |  |  |  |
|  |  |  |  |  |
|  |  |  |  |  |

Full step-by-step model building and diagnostic statistics were provided to the reviewers during the peer-review process and are available upon request.

| **Table S5. Stepwise multivariable regression analysis of LVMI as a dependent variable in male population (n=565)** | | | | |  |
| --- | --- | --- | --- | --- | --- |
|  | **Independent Contributors** | **Standardized Coefficient β** | **p-value** | **VIF** |  |
| **Model 1** | Age | 0.360 | <.001 | 1.01 |  |
| *(Adj. R² = 0.136)* | BMI | 0.112 | 0.022 | 1.01 |  |
|  | Triglycerides | -0.104 | 0.032 | 1.01 |  |
|  |  |  |  |  |  |
| **Model 2** | Age | 0.307 | <.001 | 1.10 |  |
| *(Adj. R² = 0.135)* | Antihypertensive Rx | 0.142 | 0.005 | 1.10 |  |
| Model 1: Primary clinical model. Variables excluded (p > 0.05): UHR, SBP, DBP, HbA1c, FPG, FPI, Smoking habit, Antihypertensive and Lipid-lowering therapies. Model 2: Sensitivity model adjusting for pharmacological treatments. Variables excluded (p > 0.05): UHR, SBP, DBP, BMI, Triglycerides, HbA1c, FPG, FPI, Smoking habit and Lipid-lowering therapy. Abbreviations: LVMI, Left Ventricular Mass Index; UHR, Uric Acid to HDL-cholesterol Ratio; BMI, Body Mass Index; VIF, Variance Inflation Factor. | | | | |  |
|  |  |  |  |  |  |
|  |  |  |  |  |  |
|  |  |  |  |  |  |
|  |  |  |  |  |  |

| **Table S6. Final Multivariate Regression Models for LVMI in Post-Menopausal Women (n=239)** | | | |  |
| --- | --- | --- | --- | --- |
|  |  |  |  |  |
| **Predictors** | **Standardized β** | **p-value** | **VIF** |  |
| **POST-MENOPAUSE (Adjusted R² = 0.085)** | | | |  |
| SBP | 0.275 | <.001 | 1.00 |  |
| **UHR** | **0.164** | **0.033** | **1.00** |  |
| Model based on stepwise selection. Variables excluded (p > 0.05): Age, BMI, DBP, Triglycerides, HbA1c, FPG, FPI, and Smoking habit. Age was excluded by the algorithm at Step 2 as it did not contribute significantly to the variance in this specific subgroup (where the age range is more homogeneous). Abbreviations: LVMI, Left Ventricular Mass Index; UHR, Uric Acid to HDL-cholesterol Ratio; SBP, Systolic Blood Pressure; VIF, Variance Inflation Factor. | | | |  |
|  |  |  |  |  |
|  |  |  |  |  |
|  |  |  |  |  |
|  |  |  |  |  |
